# Disorders of the cMyb proto-oncogene expression and its significance in the course of ATL development

**DOI:** 10.1186/1742-4690-11-S1-P94

**Published:** 2014-01-07

**Authors:** Kazumi Nakano, Atae Utsunomiya, Kazunari Yamaguchi, Kaoru Uchimaru, Toshiki Watanabe

**Affiliations:** 1 Graduate School of Frontier Sciences, The University of Tokyo, Tokyo, Japan; 2 Department of Hematology, Imamura Hospital Bun-in, Kagoshima, Japan; 3 Department of Safety Research on Blood and Biologics, National Institute of Infectious Diseases, Tokyo, Japan; 4 Department of Hematology and Oncology, Research Hospital, Institute of Medical Science, The University of Tokyo, Tokyo, Japan

## 

Accumulation of genetic disorders in HTLV-1 infected cells underlies ATL leukemogenesis, yet the actual genetic events responsible for cellular transformation have not been fully elucidated. Based on gene expression profiling in 52 ATL patients, 40 HTLV-1 carriers, and 21 healthy volunteers, we determined several potential risk-indicator genes of ATL, including cMyb. cMyb is the proto-oncogene of vMyb, the oncoprotein of avian myeloblastosis virus, governing hematopoietic cell differentiation. Required for differentiation of DN3, survival of DP, and generation of CD4^+^-SP cells, cMyb is not expressed at a detectable level in mature T-cells. Among well-known 7 isoforms, cMyb-9A and -10A, lacking the *cis*-acting negative regulatory domain (NRD) same as vMyb oncoprotein, are known to be molecules of “gain of oncogenic function”. We demonstrated that the mRNA levels of *cmyb-9a* and -*10a* were drastically elevated in ATL cells. Moreover, cMyb-9A protein was overexpressed in PBMC of HTLV-1 carriers and ATL patients. cMyb-9A showed the highest transactivation of HTLV-1 LTR, which is one of the cMyb targets, among 7 isoforms. The level of cMyb is known to be regulated by SUMOylation through the NRD. As expected, SUMOylation assay showed that cMyb-9A was not effectively SUMOylated, and its activity was not suppressed. Finally, cMyb-9A exhibited a significantly higher transforming activity than WT-cMyb. Upon confirming that cMyb-9A is released from the negative-regulatory circuit of cMyb, we speculate that overexpression of cMyb-9A in HTLV-1 infected cells has a strong link to disorders in cellular homeostasis by overruling its target gene expression, thus accelerating transformation process to ATL.

